# Supplementary material for: Dopamine D2 Long Receptors Are Critical for Caveolae-Mediated α-Synuclein Uptake in Cultured Dopaminergic Neurons
Source: Biomedicines. 2021 Jan 8;9(1):49. doi: 10.3390/biomedicines9010049 (PMC7826971; doi:10.3390/biomedicines9010049)
Supplement: Supplementary file 1 [file biomedicines-09-00049-s001.pdf]

Supplementary Materials:

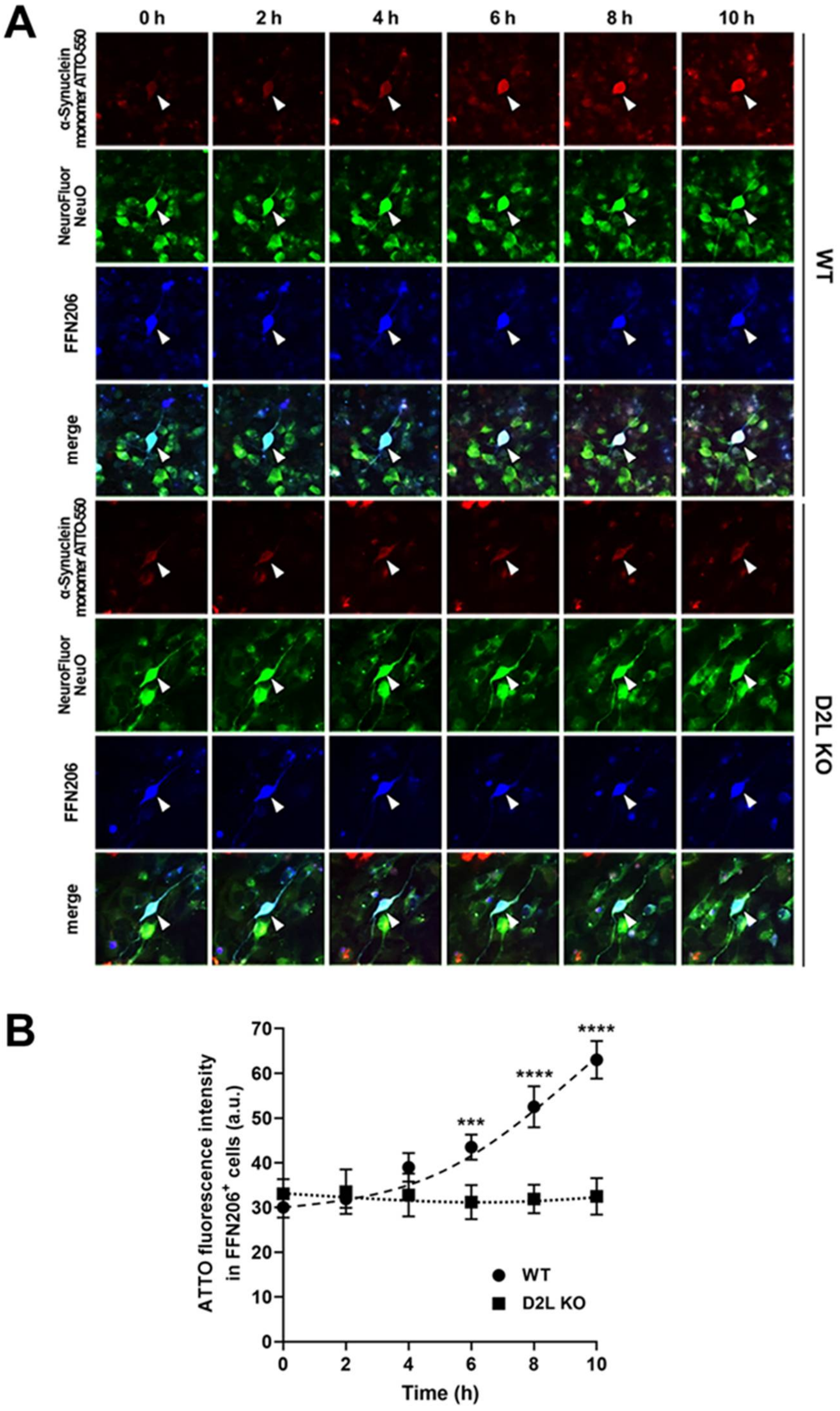

Supplementary Figure 1. Time-dependency of α-synuclein monomer uptake in dopaminergic neurons. (**A**) Wild type (WT) or dopamine $D_{2L}^{-/-}$ neurons were labeled with NeuroFluor NeurO, a neuronal marker, and FFN206, a VMAT2 substrate to indicate dopaminergic neurons, then exposed to α-synuclein monomer ATTO-550. The ability of fluorescence-labeled α-synuclein was analyzed by time-lapse imaging. (**B**) The quantitative analysis of ATTO-550-labeled α-synuclein monomer fluorescence intensity of individual $NeuO^+$ $FFN206^+$ neurons. *** $p < 0.001$, **** $p < 0.0001$ versus WT, n = 21 in three independent experiments.
